# Public health round-up

**DOI:** 10.2471/BLT.22.010922

**Published:** 2022-09-01

**Covering children’s HIV treatment needs**

A 13-year-old girl with human immunodeficiency virus (HIV) learns about the health effects of missing her antiretroviral medication from a member of the Young People and Adolescent Peer Support (YAPS) programme in Lira City, Uganda. YAPS aims to improve the quality of HIV care and increase adoption and retention of young people in need of treatment. According to a new report published by the Joint United Nations Programme on HIV/AIDS (UNAIDS) only half of children with HIV receive antiretrovirals, compared with around three quarters of adults.

**Figure:**
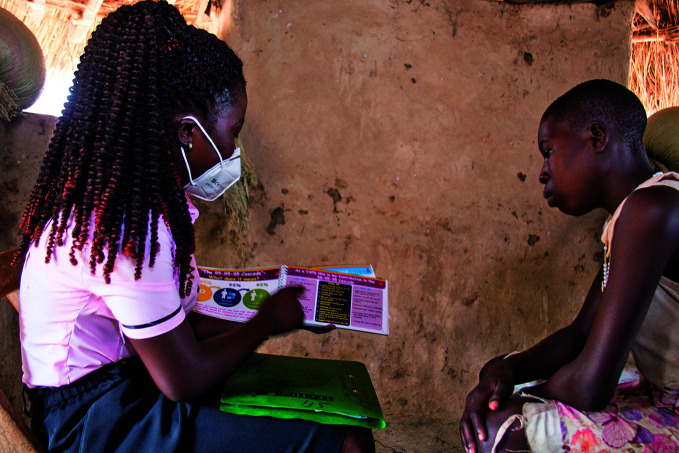
UNICEF/Jimmy Adriko

## HIV response falters

The global response to HIV has faltered with an estimated 1.5 million new infections occurring in 2021 – more than 1 million more than the global targets set.

According to a report published by the Joint United Nations Programme on HIV/AIDS (UNAIDS) on 27 July, an estimated 650 000 people died from HIV-related causes in 2021, while the number of people on HIV treatment grew more slowly than it has in over a decade.

The report cites multiple challenges to progress, ranging from the COVID-19 pandemic – which has reduced access to essential services – to the mass displacements of people due to hunger and conflict. However, lack of commitment to targets has also played a role.

“We need urgent action now and a full recommitment to our goals,” said Meg Doherty, Director of the World Health Organization’s (WHO) global HIV, hepatitis and sexually transmitted infections programmes, on the eve of the 24th International AIDS Conference which took place in Montreal, Canada between 29 July and 2 August. Doherty noted that 10 million people who are living with HIV have not yet started treatment.

https://bit.ly/3JIO6pA

## Boosting children’s HIV treatment coverage

UNAIDS, the United Nations Children’s Fund and WHO came together in a new global alliance to close the gap between HIV treatment coverage for adults and children. The new Global Alliance for Ending AIDS in Children by 2030 was announced on 2 August at the International AIDS Conference held in Montreal, Canada.

According to the above-cited UNAIDS report, only around half of children living with HIV are receiving antiretrovirals, compared with around three quarters of adults, and this disparity is increasing.

In addition to the United Nations agencies, the alliance includes civil society movements, national governments in the most affected countries, and international partners.

Twelve countries have joined the alliance in the first phase which will run until 2030.

https://bit.ly/3SHrUk0

## HIV medication licensing agreement

Specialist pharmaceutical company ViiV Healthcare and the Medicines Patent Pool signed a voluntary licensing agreement for patents relating to cabotegravir long-acting (CAB-LA) for HIV pre-exposure prophylaxis (PrEP).

CAB-LA is an intramuscular injectable, long-acting form of PrEP, with the first two injections administered 4 weeks apart, followed thereafter by an injection every 8 weeks. Shown to be safe and highly effective, CAB-LA is not yet widely available.

Under the agreement, which was announced on 28 July, selected manufacturers will be able to develop, manufacture and supply generic versions of CAB-LA in 90 countries, subject to regulatory approvals.

WHO applauded the agreement and on 28 July published new guidelines which recommend that the drug be administered by injection once every two months, as a safe and effective alternative to oral PrEP.

https://bit.ly/3p4OvcJ

https://bit.ly/3BVRs6M

## Supporting new malaria vaccine roll-out

Gavi, the Vaccine Alliance opened a process for countries to apply for funding and support to roll out the RTS,S/AS01 malaria vaccine.

Opened on 20 July, the application window follows WHO’s recommendation for wider routine use of the vaccine in October 2021 and a subsequent decision by the Gavi Board in December 2021 to approve an initial investment of US$ 155.7 million to support roll-out in the 2022–2025 period. Malaria vaccination was additionally supported by a US$ 56 million investment through an agreement with pharmaceutical company GlaxoSmithKline and a UK-based social finance company, MedAccess.

In recognition of the technical requirements of roll-out and the need to provide tailored support to countries, the first application window, which closes on 13 September 2022, will be limited to the three countries that have taken part in the vaccine’s multi-year pilot programme: Kenya, Ghana and Malawi. To date, an estimated 1.3 million children have received the vaccine in the three pilot countries.

A second window, which opens at the end of the year and closes in January, is open to other countries with moderate to high transmission of *Plasmodium falciparum* malaria. Those countries can already submit expressions of interest during the first funding window.

https://bit.ly/3PpR6bZ

## Responding to hunger in Horn of Africa

WHO launched an appeal for US$ 123.7 million on 2 August to support emergency response in the greater Horn of Africa.

Over 80 million people in the seven countries spanning the region – Djibouti, Ethiopia, Kenya, Somalia, South Sudan, Sudan and Uganda – are estimated to be food insecure, close to half of them having reached a point where they have to sell their possessions in order to feed themselves and their families.

“Hunger is a direct threat to the health and survival of millions of people in the greater Horn of Africa, but it also weakens the body’s defences and opens the door to disease,” said WHO Director-General Tedros Adhanom Ghebreyesus, calling on the international community to support WHO’s work on the ground.

The funds will go towards urgent measures to protect lives, including the provision of treatment to sick and severely malnourished children, improved national government capacity to detect and respond to disease outbreaks, procuring supplies of medicines and equipment, and identifying and filling gaps in health care.

https://bit.ly/3pjLS6T

**Cover photo**

Blood bags at the Department of Clinical Immunology, Rigshospitalet in Copenhagen, July 2021.

**Figure:**
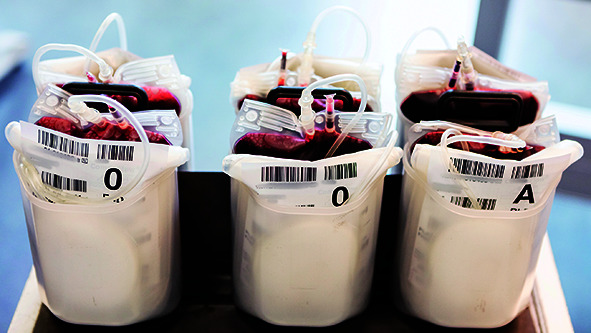
WHO/Uka Borregaard

## WHO updates COVID-19 vaccination strategy

WHO published an update to its *Strategy to achieve global Covid-19 vaccination by mid-2022,* which set out the actions required by the global community to vaccinate 70% of the world's population against COVID-19 by that date.

Published 22 July, the update is designed to reflect epidemiological changes, advancements in vaccine development and evidence, and alterations in the global vaccine programme and geopolitical landscape.

Despite being the largest and fastest in history, the global COVID-19 vaccination roll-out did not meet the mid-2022 target, and many of those at greatest risk remain unprotected. It is estimated, for example, that only 28% of older people and 37% of health care workers in low-income countries have received their primary course of vaccines and most have not received booster doses.

The updated strategy sets out adjusted global goals, steps, targets, and operational priorities to guide countries, policy-makers, civil society, manufacturers and international organizations in their ongoing efforts through 2022.

Towards the end of the year, as more scientific uncertainties get resolved and more data becomes available, WHO will embark on a consultative process to develop a global COVID-19 vaccination strategy for 2023 and beyond.

https://bit.ly/3w4nf1Y

## Pandemic prevention instrument takes shape

The Intergovernmental Negotiating Body (INB) tasked with drawing up a new international instrument on pandemic prevention, preparedness and response concluded its second meeting, with an agreement to work on an instrument that will be legally binding.

In a 21 July statement, WHO Director-General Tedros Adhanom Ghebreyesus welcomed the development saying, “The importance of a legally binding instrument cannot be overstated: it will be our collective legacy for future generations.”

The INB is a subdivision of the World Health Assembly, the decision-making body of WHO and is comprised of WHO’s 194 Member States, Associate Members, and regional economic integration organizations. INB members will work to conclude the agreement by May 2024.

https://bit.ly/3w6MG2R

## Renaming monkeypox variants

Experts convened by WHO on 8 August agreed on new names for monkeypox virus variants, as part of ongoing efforts to ensure that the names of the monkeypox disease, virus and variants – or clades – reflect current best practice.

That practice includes the use of names that avoid offending any cultural, social, national, regional, professional or ethnic groups, and minimize any negative impact on trade, travel, tourism or animal welfare.

The experts agreed to name the clades using Roman numerals to identify the former Congo Basin (Central African) and West African clades as Clade I and II, respectively.

The naming of the virus and associated disease is the responsibility of the International Committee on Taxonomy of Viruses which has launched a process to arrive at suitable terminology.

https://bit.ly/3QptqFR

**Looking ahead**

1–2 September, H20: Annual meeting of the G20 health ministers’ group. https://bit.ly/3uE39L6

4–7 September, 23rd IUSTI World Congress – Confronting Inequities in STI Prevention, Diagnostics and Care. https://bit.ly/3QDw4HD

19–23 September, Global conference on Violence Against Women and Children. https://bit.ly/3QmGbAS

